# Explainability Methods from Machine Learning Detect Important Drugs’ Atoms in Drug-Target Interactions

**DOI:** 10.1021/acs.jcim.6c00037

**Published:** 2026-04-15

**Authors:** Mrinal Mahindran, Qingyuan Liu, Vishak Madhwaraj Kadambalithaya, Olga V. Kalinina

**Affiliations:** † Center for Bioinformatics, 9379 Saarland University, Saarbrücken 66123, Germany; ‡ Helmholtz Institute for Pharmaceutical Research Saarland (HIPS), 28336 Helmholtz Centre for Infection Research (HZI), Saarbrücken 66123, Germany; § International Max Planck Research School on Trustworthy Computing, Saarbrücken 66123, Germany; ∥ Medical Faculty, Saarland University, Homburg 66421, Germany

## Abstract

Predicting drug-target interactions (DTI) with graph neural networks (GNNs) is hindered by their lack of interpretability. To address this, we benchmark four explainable artificial intelligence (XAI) attribution methods on GNN models trained for kinase and G-protein-coupled receptors (GPCR) targets. We assess the methods’ consistency through atom-level intersection over union (IoU) and validate their biological relevance by mapping attributed atoms to three-dimensional (3D) protein–ligand structures. While consistency across methods was modest, consensus attributions were highly enriched for atoms directly contacting the binding pocket—up to 76% within 2 Å in the kinase-inhibitor complexes. Notably, these attributed atoms were frequently found contacting experimentally important regulatory residues such as those in the DFG motif. This indicates that XAI methods, despite their disagreements, can identify chemically meaningful ligand features, providing a foundation for developing more interpretable GNNs in drug discovery.

## Graphical abstract

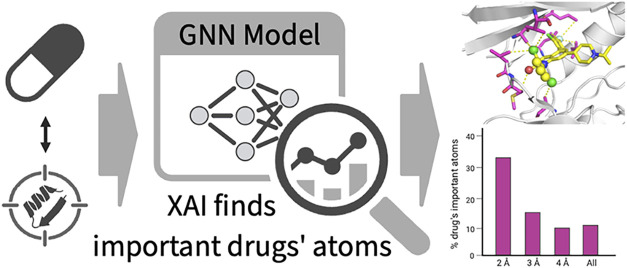

## Introduction

In drug discovery, identifying viable drug candidates from a vast library of molecules presents a profoundly complex challenge.^1,2^ Computational drug-target interaction (DTI) prediction methods have become essential tools to bridge the gap between theoretical exploration of the chemical space and resource-intensive experimental validation (e.g., high-throughput screening).^3,4^ Currently, deep learning approaches represent the most widely adopted methods among computational DTI prediction techniques, demonstrating significant utility for enhanced binding affinity prediction, off-target identification, and drug repurposing, among other critical applications in modern-day drug discovery, especially the early stages of drug discovery.^5–7^

Recent advances in deep learning, especially graph neural networks (GNNs), have developed DTI predictions by representing proteins and ligands as graphs, capturing complex chemical and topological dependencies through message-passing frameworks.^8–14^ Among the corresponding architectures, GNNs have demonstrated superior ability in modeling molecular interactions despite higher computational demands compared to classical machine learning or deep learning models.^15,16^ Their strength lies in approximating highly nonlinear structure–activity relationships directly from raw graph representations of chemical structures,^17,18^ potentially superseding classic hand-crafted molecular fingerprints.^17^ Nevertheless, adoption of GNNs in drug discovery remains limited due to their “black-box” nature and the inability to provide chemically intuitive explanations for predictions.^2,19^ This interpretability gap is further compounded by risks such as the Clever Hans effect: producing correct answers for the wrong reasons,^20^ and overconfident erroneous outputs.^21^ Explainable artificial intelligence (XAI) methods aim to address these issues by making GNN decision processes transparent^22–26^ —revealing which substructural motifs in the ligand and which residue interactions in the protein drive the predicted binding—thus bridging machine reasoning with chemical intuition.^2,27–30^

Explainable AI (XAI) methods can be broadly classified into attribution-based and non-attribution-based approaches. Attribution-based techniques—often described as feature-importance methods—aim to identify which input features contributed most to a model’s prediction;^31^ they typically produce “heatmaps” of importance but do not reveal the decision logic or offer actionable guidance for molecular design.^32^ By contrast, nonattribution methods employ logical rules, comparative examples, or exploit intrinsic model structure to produce explanations that better align with human reasoning and chemical intuition.^32^

Many rule- or example-based explainers^33^ and some attribution approaches^24^ rely on perturbing model inputs. For DTI tasks, perturbation-based strategies often introduce unnatural artifacts^34^ or chemically invalid subgraphs,^35^ thereby shifting inputs into out-of-distribution regions.^36^ Such perturbations can fragment aromatic rings or essential pharmacophores, degrading structural integrity and producing unfaithful explanations. Graph-specific XAI methods (e.g., GNNExplainer,^26^ SubgraphX,^37^ PGExplainer^38^) instead target important subgraphs—identifying core motifs or substructures that contribute to binding—but they frequently suffer from instability and adversarial vulnerability, limiting their reliability in practice.^32^ Other alternatives have practical limitations: CXplain^39^ requires retraining an auxiliary model, which is computationally expensive and difficult to stabilize for graph neural networks.^35,40^

Multiple existing studies have addressed the necessity to provide interpretations for predictions primarily through the visualization of attention weights.^41,42^ While the application of attention mechanisms is straightforward and effective, it remains limited to attention- or transformer-based GNN architectures and thus cannot be uniformly applied to diverse network architectures. Furthermore, such mechanisms typically capture only local vertex neighborhoods (often termed masked attention),^43,44^ failing to elucidate global relationships between atoms.^45^

Gradient-based XAI methods offer greater universality across diverse GNN architectures and have demonstrated considerable promise.^45^ These techniques—such as Input × Gradient,^46^ Guided Backpropagation (GB),^47^ Integrated Gradients,^48^ and Gradient SHAP^49^—are broadly applicable to deep learning models and therefore offer a more universal option for generating attributions without explicit architectural constraints. To our knowledge, however, no comprehensive benchmark exists for evaluating gradient-based attribution methods, specifically on DTI GNN models. To address this gap, our study implements and assesses four attribution-based XAI techniques: Input × Gradient, Guided Backpropagation, Integrated Gradients, and Gradient SHAP. We apply these to GNN-based DTI models trained on two curated data sets comprising interactions of two distinct protein families, G-protein-coupled receptors (GPCRs)^50^ and protein kinases,^4,51^ with low-molecular-weight ligands. We trained the models in four different data splitting regimes: random, cold-drug, cold-target, and scaffold, along with an additional biological relevance split, and then applied the attribution methods to the best-performing model for analysis. We introduce complementary assessments: first, quantifying the explanation agreement through intersection-overunion (IoU) of attributed atoms; and second, validating biological relevance by mapping consensus-attributed atoms against known binding site residues and pharmacophoric features. This work presents a systematic framework for benchmarking the consistency of attribution and biological plausibility in GNN explanations for DTI.

## Methods

The overall experimental framework—encompassing data preparation, GNN architectural evaluation, and post hoc attribution analysis—is visually summarized in Figure 1.

**Figure 1:**
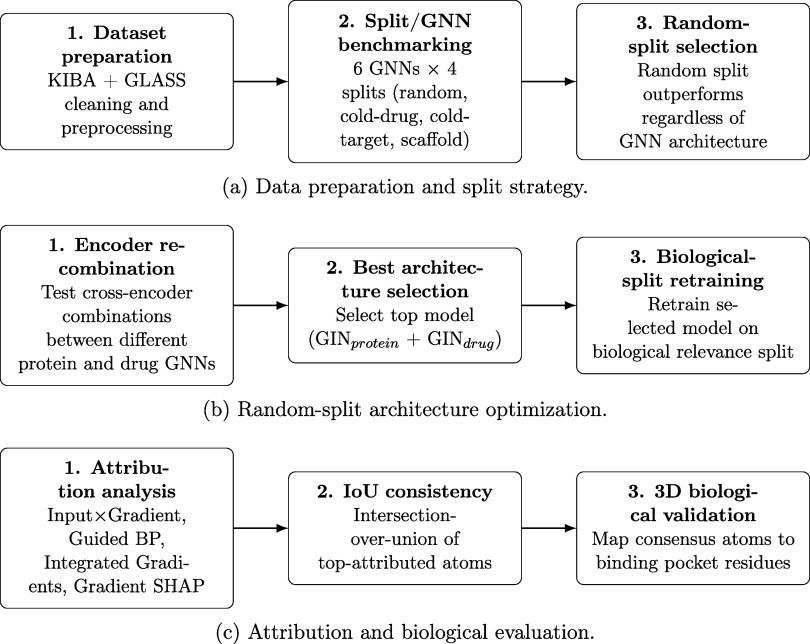
Overview of the experimental framework for graph neural network attribution analysis. (a) Data preparation and split strategy selection: The KIBA and GLASS data sets underwent rigorous cleaning and preprocessing and were then encoded as molecular graphs. For small molecules, we used RdKit,^52^ and for proteins we used a distance-based cutoff of 8 Å to identify edges between vertices corresponding to amino acid residues as implemented in the RINDTI package. Six distinct GNN architectures (Graph Isomorphism Network (GINConv or GIN), GraphSAGE, Graph Attention Network (GATConv), Transformer-based Graph Network, FiLMConv, and ChebConv) were evaluated across four data splitting methodologies (random, cold-drug, cold-target, and scaffold) to identify baseline predictive limits. The random split was ultimately selected for subsequent steps due to the superior baseline performance across all architectures. (b) Random split architecture optimization: Fixing the data split, cross-encoder combinations (independently pairing different GNN families for the protein and ligand branches) were tested. The dual Graph Isomorphism Network (GIN_protein_ + GIN_drug_) provided the strongest continuous prediction and was subsequently retrained exclusively on a specialized, structurally verified biological relevance data split. (c) Attribution and biological evaluation: The optimized model was probed post hoc using four gradient-based explainable AI (XAI) methods (Input × Gradient, Guided Backpropagation, Integrated Gradients, and Gradient SHAP). The resulting individual atom importance scores were assessed for consistency via an Intersection-over-Union (IoU) consensus threshold, and the most heavily attributed consensus atoms were mapped directly to true three-dimensional (3D) spatial binding pocket residues for physical validation.

### Data Sets and Preprocessing

We evaluated our models on two widely used bioactivity data sets: the KIBA data set of kinase inhibitors^4^ and the GLASS data set of the association of GPCR-ligands.^50^ The KIBA and GLASS data sets represent two critical resources in drug discovery, each focusing on a therapeutically pivotal protein family.

The KIBA data set focuses on kinases, representing a critical protein family that, among other functions, regulates signaling pathways in eukaryotic cells,^53^ making them prime drug targets in oncology and immunology.^53^ To describe interactions between kinases and the corresponding inhibitors, the KIBA score integrates three binding metrics (*K_i_*, *K_d_*, IC_50_) into a unified bioactivity value, enabling robust prediction of compound efficacy against kinase subfamilies (e.g., tyrosine kinases).^4^ The data set contains 246,088 KIBA scores across 52,498 chemical compounds and 467 kinase targets. The lower KIBA score indicates a higher binding affinity between the kinases and the drugs. For training our models, we removed any drug or target with fewer than 10 total recorded values in the data set. KIBA scores were then negated and shifted to set the minimum value to zero, preserving bioactivity rankings while ensuring a non-negative regression target.

The GLASS data set focuses on G-protein-coupled receptors (GPCRs), a protein family crucial in drug discovery due to their role in mediating diverse physiological processes (e.g., neurotransmission, immune responses)^54^ and their status as targets for ∼35% of FDA-approved drugs.^54^ The GLASS data set was first cleaned by removing any interactions that did not have a reference to any database or publication, or whose references were broken links, as their validity could not be confirmed. Any interactions with invalid energy scores (recorded values are not a number) were also discarded. Furthermore, because of the compilation of interactions from various data sets, even after removing entries with the same value and references, some duplicate interactions bypassed filters. The difference between such duplicates was the difference in rounding. Such entries were manually checked and then removed. The newly cleaned data set contains 513,246 unique compound–GPCR pairs (681 GPCRs and 277,651 ligands), down from the original 562,871 pairs. We retained only those entries with explicitly reported *K_i_* values, which were all transformed to their corresponding p*K_i_* values and scaled by a factor of 10^–9^ for stabilizing model training^5^ using p*K_i_* = −log_10_(*K_i_* × 10^–9^).

For both data sets, SMILES strings were processed using RDKit^52^ to generate molecular structures, which were further canonicalized and sanitized to ensure consistent atom ordering. Molecular graphs were produced with PyTorch Geometric.^55^ Compounds with more than 150 heavy atoms were excluded to reduce computational overhead, and data points with missing SMILES or affinity values were discarded. The remaining number of data points is summarized in Table 1.

**Table 1:** Summary of Data Sets and Binding Affinity Scores in the Clean Data Sets after Filtering

data set	proteins	ligands	data points	binding score
GLASS	490	70,517	136,085	p*K_i_* (log *K_i_*)
KIBA	421	2353	148,327	KIBA score (normalized)

### Graph Neural Networks

Message-passing neural networks (MPNNs), a class of graph neural networks (GNNs), form the foundation of our architecture. In this framework, both drug molecules and proteins are represented as graphs *G* = (*V*, *E*). The molecular graph representation for each drug is constructed from its SMILES string mapped into the undirected graph *G_D_* = (*V_D_*, *E_D_*). The nodes are characterized by feature vectors $x_{D}\in R^{d_{D}}$ representing one-hot encoded atomic types, while the edges are determined by the molecular bonding topology. Similarly, each protein is represented as a residue-level graph *G*_*P*_ = (*V*_*P*_, *E*_*P*_) derived from its Protein Data Bank (PDB) structure. Here, node features $x_{P}\in R^{d_{P}}$ are one-hot encodings of the 20 standard amino acid types, and an edge is created between two residues if the distance between their α-carbons (Cα) is less than 8 Å.

The MPNN operates through a series of iterative message-passing layers that update node representations by aggregating information from their local neighborhoods. At each layer *k*, the process involves two main steps. First, in the message aggregation step, each node *v* collects messages from its neighbors N(v) according to

1 $$m_{v}^{\left(k\right)}=\sum_{u\in N\left(v\right)}\phi \left(h_{u}^{\left(k-1\right)},e_{\mathrm{uv}}\right)$$

where *h*_*u*_^(*k*–1)^ is the feature vector of a neighboring node *u* from the previous layer, *e*_*uv*_ is the feature vector of the edge between them, and ϕ is a learnable message function. Second, in the feature update step, the node’s representation is updated by combining its previous state with the aggregated message

2 $$h_{v}^{\left(k\right)}=\psi \left(h_{v}^{\left(k-1\right)},m_{v}^{\left(k\right)}\right)$$

where ψ is an update function, typically a neural network with a nonlinear activation function such as ReLU. This message-passing scheme is repeated for *K* layers, allowing the model to progressively capture information from larger structural contexts. The final graph-level representations are then used to predict drug-target interactions via downstream neural network architectures. Both the message function ϕ and the update function ψ contain learnable parameters that are optimized end-to-end during training by using backpropagation.

### Model Architecture and Training

The model uses a dual-encoder framework where separate graph neural networks (GNNs) encode the ligand and protein graphs. Each encoder processes its respective graph to produce a fixed 256-dimensional vector representation.

We experimented with several fundamental GNN variants including graph isomorphism network (GINConv or GIN),^56^ GraphSAGE (graph sample and aggregate),^57^ graph attention network (GATConv),^43^ transformer-based graph network (Transformer-GNN),^58^ feature-wise linear modulation convolution (FiLMConv),^59^ and Chebyshev convolution (ChebConv),^60^ as implemented in the package RINDTI (https://github.com/kalininalab/rindti). We explicitly chose to build our models around these architectures using purely generic atom-level embedding to prevent the network from relying on precalculated, human-engineered structural fingerprints, which many state-of-the-art models rely on. Simple GNN models directly map basic node embeddings back to the corresponding structural features, guaranteeing that our XAI techniques specifically track geometric importance at the atomic level. Furthermore, employing separate GNNs natively on both the protein and the ligand ensures spatial representation is learned intrinsically rather than forced.

Each encoder applied multiple message-passing layers, followed by mean pooling to obtain a graph-level embedding. The resulting ligand and protein embeddings were then concatenated and passed to a multilayer perceptron (MLP) for affinity prediction. Models were trained using mean squared error (MSE) loss and optimized with Adam^61^ (learning rate equal to 0.0001) with a batch size of 256, except for attention-based architectures, which were trained with a batch size of 128. Training was conducted for up to 500 epochs with early stopping triggered after 50 epochs without a validation loss improvement. All experiments were implemented using PyTorch Geometric, and random seeds were fixed for reproducibility (see Supporting Note 2 and Table S1 in the Supporting Information for a complete summary of the architectural and training details).

### Data Splitting Strategies

To comprehensively evaluate the model generalization, we employed four distinct data splitting strategies. The baseline approach was a *random split*, in which the data set was randomly divided into 80% training, 10% validation, and 10% test sets. This splitting scenario implies that the validation and test sets contain the same drugs and proteins seen during training and thus represents the least challenging scenario. For more rigorous testing, we utilized two *cold* splits. The *cold drug split* ensured that all drug molecules in the validation and test sets were completely unseen during training, a method designed to evaluate how well the model generalizes to novel compounds. Alternatively, the *cold target split* ensured that proteins in the test set were absent from the training set. This approach assesses the model’s ability to infer binding interactions for unseen biological targets, a crucial capability for applications such as drug repurposing, and is empirically considered one of the most challenging evaluation scenarios in DTI modeling. Additionally, a *scaffold split* was performed, partitioning compounds by their Bemis–Murcko scaffolds to ensure structural dissimilarity among the training, validation, and test sets. Furthermore, to assess the biological plausibility of model attributions, we designed a separate *biological relevance split*, which consisted of moving all drug–target pairs with available protein–ligand complex structures into the test set to enable a direct structure-based evaluation. This split was used solely for biological analysis and did not influence training (i.e., hyperparameter tuning, etc.) and selection of the GNN models.

### Cross-Data Set Fine-Tuning

To assess the transferability of learned representations, we performed a targeted cross-data set fine-tuning procedure. For each transfer direction (KIBA → GLASS and GLASS → KIBA), we initialized from the best dual-encoder checkpoint on the source data set (selected by the lowest test MSE). The encoder parameters, including all message-passing layers of both the drug and protein branches, were frozen. At the same time, the downstream multilayer perceptron (MLP) prediction head was reinitialized and trained on the training partition of the target data set. Fine-tuning employed the same optimization and regularization settings as those in the main experiments to ensure comparability.

### Attribution Methods

This section details the feature attribution techniques implemented to interpret the predictions of our graph neural network model for the drug-target binding affinity. We evaluate four gradient-based methods: Input × Gradient,^46^ Guided Backpropagation,^47^ Integrated Gradients,^48^ and Gradient SHAP.^49^ Each method assigns importance scores, $S_{v}\in R$ to atoms *v* ∈ *V* in the molecular graph, quantifying their contribution to the predicted binding affinity *F*(*G*_*D*_, *G*_*P*_).

#### Attribution Framework

Formally, our trained graph neural network model is represented as a function *F*(*x*) that outputs drug-target binding affinity predictions, where *x* denotes the input feature matrix encoding both the drug molecular graph and the protein structure. A feature attribution method computes importance scores for individual input features, specifically the atoms in the drug molecular graph. The attribution function *A* assigns a score *S*_*i*_ to each atom feature *x*(*i*) according to the following relationship

3 $$S_{i}=A\left(F,x,i\right)$$

Here, *S*_*i*_ represents the attribution score for the *i*-th atom, quantifying its contribution to the final binding affinity prediction *F*(*x*). The function *A* denotes the specific attribution method used, such as Input × Gradient or Integrated Gradients.

#### Input × Gradient

The Input × Gradient (I × G) method computes importance scores by calculating the element-wise product of the input feature values and the gradient of the model’s output with respect to those features. The attribution score is formally defined as

4 $$S_{i_{\mathrm{Input}\times \mathrm{Gradient}}}=x\left(i\right)⊙\frac{\partial F\left(x\right)}{\partial x\left(i\right)}$$

where *x*(*i*) is the input feature of *i*-th atom and $\frac{\partial F\left(x\right)}{\partial x\left(i\right)}$ is the gradient of the model output *F*(*x*) with respect to that feature.

#### Guided Backpropagation

The Guided Backpropagation (GB) method further refines the Input × Gradient method by propagating only positive gradients through ReLU activation. This filtering mechanism is applied to $\frac{\partial F\left(x\right)}{\partial x_{i}}$ , effectively setting gradients to zero if the gradient is negative. This process is intended to reduce the noise in the resulting attributions. The final score is then calculated by using the filtered gradient

5 $$\frac{\partial F\left(x\right)}{\partial x_{i}}=\{\begin{array}{ll} \nabla F\left(x\right) & \mathrm{if}\;\nabla F\left(x\right)>0\;\mathrm{and}\;x>0\\ 0 & \mathrm{otherwise} \end{array}$$

6 $$S_{i_{\mathrm{Guided}\;\mathrm{Backpropagation}}}=x\left(i\right)⊙\frac{\partial F\left(x\right)}{\partial x\left(i\right)}$$

#### Integrated Gradients

The Integrated Gradients (IG) method computes feature attributions by accumulating gradients along a path from baseline input *x*′ (typically a zero vector) to actual input *x*. The method defines a straight-line path *x*(*t*) = *x*′ + *t* · (*x* – *x*′) for *t* ∈ [0, 1]. The attribution is the path integral of the gradients with respect to the input features, scaled by the difference between the input and the baseline

7 $$S_{i_{\mathrm{IG}}}=\left(x-x^{\prime }\right)⊙\int_{0}^{1}\nabla F_{i}\left(x\left(t\right)\right)dt$$

In practice, this integral is approximated numerically by summing the gradients over small intervals along the path.

#### Gradient SHAP

Gradient SHAP extends the Integrated Gradients framework by improving the choice of the baseline. Instead of using a single, fixed baseline, it computes the expectation of the IG attributions over a distribution of baselines, *P*(*x*′). This is typically implemented by sampling multiple baselines {*b*_1_, *b*_2_, ···, and *b*_*S*_}, often by adding small Gaussian noise to a zero vector, computing the IG attribution for each, and averaging the results

8 $$SHAP\left(x\right)=E_{x^{\prime }∼P\left(x^{\prime }\right)}\left[IG\left(x,x^{\prime }\right)\right]$$

These attribution methods were applied exclusively to the ligand graph, yielding a single attribution score for each atom after processing.

#### Attribution Processing

For atomic-level interpretation, the raw feature-level attribution scores were postprocessed. First, for each atom *v*, the scores across all of its feature dimensions were aggregated into a single score, *S*_*v*_, by taking their sum. Second, to reduce noise and enhance visual clarity, we effectively discarded any atomic scores where the absolute value of |*S*_*v*_| was less than 10^–4^.

### Validation of Biological Relevance

To assess whether the most highly attributed atoms correspond to biologically meaningful regions, we analyzed their spatial proximity to known protein binding pockets by using experimentally resolved ligand–protein structures. For the KIBA data set, we used the KLIFS^62^ database, which provides curated kinase–inhibitor complexes with annotated pocket residues. For GLASS, we retrieved experimentally determined ligand–protein complexes from the RCSB Protein Data Bank (PDB).

We first computed the intersection-overunion (IoU) of the top-*k*% most attributed atoms for all four explanation methods for each data point (protein–drug interaction), varying *k* across multiple thresholds (Table 2). The primary advantage of employing the IoU metric is its simple and robust implementation, allowing for a straightforward binary combination of attributions to measure consistency between disparate methods. The main disadvantage, however, is that after calculating the intersection, the continuous attribution weights are stripped; therefore, there are no remaining granular scores that can rank the inputs in order of importance within the resulting consensus subset.

**Table 2:** Intersection-Over-Union (IoU) of Important-Atom Sets at Different Top-*k* Attribution Thresholds for KIBA and GLASS

top-*k*	KIBA	GLASS
10%	0.0140	0.0141
20%	0.0304	0.0333
30%	0.0570	0.0647
40%	0.0972	0.1066
50%	0.1526	0.1636

IoU scores were averaged across all data points to obtain a data set-level measure of agreement. For downstream structural analysis, we focused on the 50% threshold and retained only those data points where (a) the attributed atoms overlapped and (b) the three-dimensional (3D) structural data were available. For each such example, we extracted the consensus-attributed atoms and copied their 3D coordinates from the MOL2 or PDB structures. We subsequently calculated the shortest Euclidean distance from every attributed atom to all atoms in the respective protein binding pocket. Moreover, we measured biological alignment by reporting the proportion of consensus atoms that were within distance cutoffs between 2 and 4 Å from the binding pocket residues. An upper limit of 4 Å is a widely accepted empirical distance cutoff for identifying direct noncovalent intermolecular interactions (e.g., hydrogen bonding, hydrophobic contacts) between a ligand and protein target. We report thresholds at 2, 3, and 4 Å primarily to illustrate the spatial trend showing how the percentage of attributed atoms drops off structurally as the distance to the pocket increases. This provided a structure-aware and stringent assessment of whether the model’s most prominent predictions overlapped with known interaction sites.

## Results and Discussion

### Model Performance

The model performance of the various GNN-based models was evaluated on both KIBA and GLASS data sets across multiple data splits, including random, cold drug, cold target, and scaffold. In addition, the best-performing model was further evaluated under the biological relevance split, which was reserved solely for downstream structural analysis. Performance was assessed using the mean squared error (MSE), root mean squared error (RMSE), concordance index (CI), and Pearson correlation coefficient between predicted and actual binding affinities to provide a comprehensive evaluation.

For both data sets, models using GINConv for both the protein and drug encoders consistently outperformed other architectures (Table 3). For the KIBA data set, the random split yielded the best performance, with the GIN_protein_–GIN_drug_ model achieving an MSE of 0.1741 (RMSE: 0.4159, CI: 0.8730, Pearson: 0.8704). Performance dropped for cold drug and cold protein splits, mirroring the greater challenge of generalization to new compounds or targets. The scaffold split, which groups molecules based on shared core structures to better test scaffold-level generalization, resulted in the poorest performance across almost all architectures for the KIBA data set (e.g., MSE = 0.5859 using GINConv). This indicates that for this data set, generalizing to fundamentally novel molecular scaffolds poses an even greater challenge than simply encountering an unseen drug or target.

**Table 3:** Performance Comparison across Different Data Splits on KIBA and GLASS^a^

	KIBA				GLASS			
model	random	cold-drug	cold-target	scaffold	random	cold-drug	cold-target	scaffold
GINConv	MSE: **0.1741**	MSE: 0.4607	MSE: 0.4725	MSE: 0.5859	MSE: **0.4766**	MSE: 0.5583	MSE: 1.4180	MSE: 0.7806
	RMSE: **0.4159**	RMSE: 0.6787	RMSE: 0.6874	RMSE: 0.7654	RMSE: **0.6897**	RMSE: 0.7472	RMSE: 1.1908	RMSE: 0.8835
	CI: **0.8730**	CI: 0.7307	CI: 0.7582	CI: 0.6653	CI: **0.8172**	CI: 0.7966	CI: 0.6221	CI: 0.7485
	P: **0.8704**	P: 0.6416	P: 0.6874	P: 0.4880	P: **0.8235**	P: 0.7855	P: 0.3734	P: 0.6883
GATConv	MSE: **0.3228**	MSE: 0.5608	MSE: 0.5774	MSE: 0.7577	MSE: **0.7942**	MSE: 0.7731	MSE: 1.4573	MSE: 1.0360
	RMSE: **0.5681**	RMSE: 0.7489	RMSE: 0.7599	RMSE: 0.8705	RMSE: **0.8914**	RMSE: 0.8793	RMSE: 1.2072	RMSE: 1.0178
	CI: **0.7878**	CI: 0.6844	CI: 0.6562	CI: 0.5849	CI: **0.7470**	CI: 0.7461	CI: 0.5682	CI: 0.6908
	P: **0.7421**	P: 0.4980	P: 0.5035	P: 0.3148	P: **0.6878**	P: 0.6856	P: 0.2241	P: 0.5536
GraphSAGE	MSE: **0.3307**	MSE: 0.5936	MSE: 0.7717	MSE: 0.7917	MSE: **0.8559**	MSE: 0.8665	MSE: 1.4935	MSE: 1.0273
	RMSE: **0.5746**	RMSE: 0.7705	RMSE: 0.8780	RMSE: 0.8898	RMSE: **0.9246**	RMSE: 0.9309	RMSE: 1.2221	RMSE: 1.0136
	CI: **0.7879**	CI: 0.6563	CI: 0.5204	CI: **0.7349**	CI: 0.5736	CI: 0.7230	CI: 0.5141	CI: 0.6830
	P: **0.7390**	P: 0.4316	P: 0.1213	P: 0.2177	P: **0.6610**	P: 0.6350	P: 0.0774	P: 0.5375
Transformer-GNN	MSE: **0.2864**	MSE: 0.5627	MSE: 0.5544	MSE: 0.6236	MSE: **0.6990**	MSE: 0.7695	MSE: 1.7665	MSE: 1.0678
	RMSE: **0.5172**	RMSE: 0.7501	RMSE: 0.7446	RMSE: 0.7897	RMSE: **0.8355**	RMSE: 0.8772	RMSE: 1.3291	RMSE: 1.0333
	CI: **0.8198**	CI: 0.6926	CI: 0.6761	CI: 0.6557	CI: **0.7643**	CI: 0.7433	CI: 0.5138	CI: 0.6797
	P: **0.7923**	P: 0.4956	P: 0.5121	P: 0.4169	P: **0.7247**	P: 0.6803	P: 0.0616	P: 0.5272
FiLMConv	MSE: **0.1877**	MSE: 0.4729	MSE: 0.4866	MSE: 0.6720	MSE: **0.5993**	MSE: 0.6523	MSE: 1.4901	MSE: 0.8536
	RMSE: **0.4335**	RMSE: 0.6877	RMSE: 0.6976	RMSE: 0.8198	RMSE: **0.7738**	RMSE: 0.8077	RMSE: 1.2207	RMSE: 0.9239
	CI: **0.8654**	CI: 0.7100	CI: 0.7251	CI: 0.6565	CI: **0.7882**	CI: 0.7734	CI: 0.5618	CI: 0.7316
	P: **0.8579**	P: 0.5879	P: 0.5793	P: 0.3985	P: **0.7710**	P: 0.7423	P: 0.1911	P: 0.6495
ChebConv	MSE: **0.4961**	MSE: 0.6623	MSE: 0.7891	MSE: 0.6194	MSE: **1.0753**	MSE: 1.0374	MSE: 1.5812	MSE: 1.1428
	RMSE: **0.7048**	RMSE: 0.8138	RMSE: 0.8883	RMSE: 0.7870	RMSE: **1.0371**	RMSE: 1.0185	RMSE: 1.2575	RMSE: 1.0690
	CI: **0.7181**	CI: 0.6632	CI: 0.5204	CI: 0.6367	CI: **0.6757**	CI: 0.6791	CI: 0.4783	CI: 0.6480
	P: **0.5572**	P: 0.4193	P: 0.0572	P: 0.3291	P: **0.5192**	P: 0.5299	P: 0.0013	P: 0.4460

a Each cell reports **MSE**↓/**RMSE**↓/**CI**↑/**Pearson**↑. Lower is better for MSE and RMSE, while higher is better for CI and Pearson. Values in bold correspond to the random split, which consistently yields the best overall performance across both data sets.

For the GLASS data set, models showed similar overall performance trends but with a distinct tiering in generalization difficulty. The GINConv-based model with random splitting achieved an MSE of 0.4766 (RMSE: 0.6897, CI: 0.8172, Pearson: 0.8235). In contrast to KIBA, the GLASS data set exhibited a clear hierarchy across the out-of-distribution splits: the scaffold split consistently yielded worse performance than the cold-drug split but maintained better performance than the cold-target split. This suggests that while generalizing to entirely new target proteins remains the single most difficult task in the GLASS data set, achieving structural generalization across novel drug scaffolds is still significantly more challenging than inferring affinities for novel (yet structurally represented) drug compounds.

We attribute this structural generalization discrepancy between the two data sets to the vast differences in their underlying chemical diversity. While the KIBA data set contains roughly ∼68% as many unique protein targets as the GLASS data set (467 vs 681), its chemical space is severely restricted, harboring less than 19% of the unique ligands available in GLASS (52,498 vs 277,651). Because a scaffold split enforces an even harsher penalty on chemical dissimilarity than a standard cold-drug split, the limited pool of unique ligands in KIBA inherently starves the model of the diverse scaffold coverage needed to successfully generalize to unrepresented chemical subspaces. Conversely, the significantly larger chemical repertoire in GLASS allows the model to map a wider array of molecular structures, thereby softening the impact of a scaffold split such that true zero-shot target generalization (cold-target) rightfully re-emerges as the most difficult prediction task.

We also explored hybrid models where different encoder architectures were used for the protein and drug graphs (Table 4). These models were trained using the random split, since this split consistently yielded the best results across both data sets in previous experiments. Even in this comparison, the GIN_protein_–GIN_drug_ model consistently outperformed all of the other configurations. To assess the models’ robustness, we performed multiseed cross-validation where all baseline GNN architectures were retrained on the random split across five different seeds (Table 5). Under this rigorous evaluation, the GIN_protein_–GIN_drug_ model maintained its superior predictive capability across both data sets. Finally, the selected GIN_protein_–GIN_drug_ model was applied to the biological relevance split to support downstream structural validation, where it achieved an MSE of 0.26 for KIBA and 0.50 for GLASS.

**Table 4:**
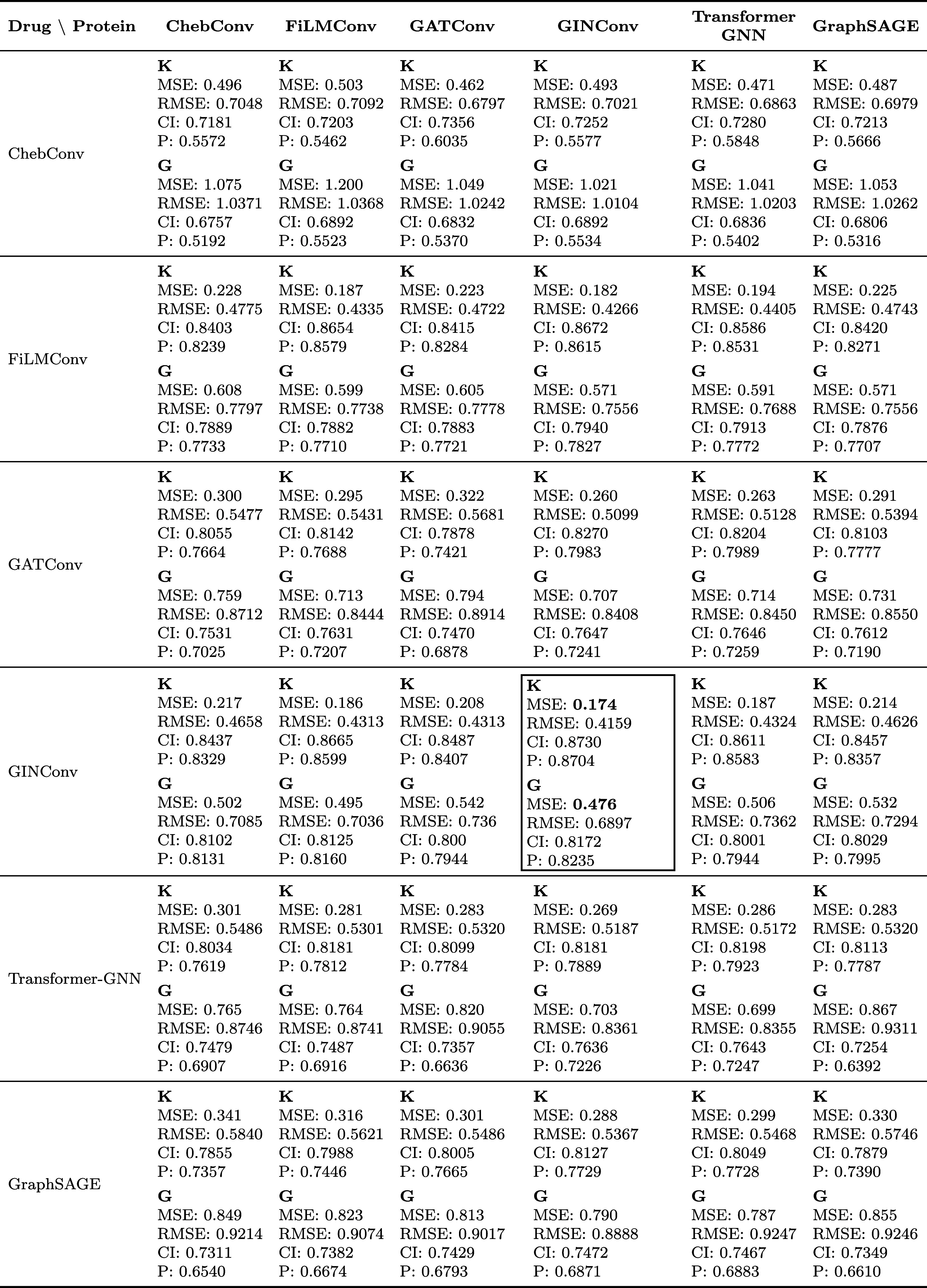
Performance Comparison of Different Drug and Protein Encoder Combinations on the Random Split^a^

a Each cell reports **KIBA (K)** and **GLASS (G)** in the order **MSE**↓/**RMSE**↓/**CI**↑/**Pearson**↑.

**Table 5:** Test MSE (Mean ± SEM over Five Seeds) for Different Graph Encoder Architectures Evaluated on the GLASS and KIBA Data Sets

encoder	GLASS	KIBA
GINConv	0.4893 ± 0.0045	0.174 ± 0.0018
ChebConv	1.0652 ± 0.0066	0.4861 ± 0.0035
GraphSAGE	0.8442 ± 0.0103	0.3295 ± 0.0009
Transformer-GNN	0.7419 ± 0.0122	0.2702 ± 0.0042
GATConv	0.7574 ± 0.0174	0.2931 ± 0.0075
FiLMConv	0.5900 ± 0.0025	0.1976 ± 0.0028

Additionally, we explored cross-data set fine-tuning, where the best model was trained on one data set and fine-tuned on the other under the random split configuration. The best model (GIN_protein_–GIN_drug_ model) trained on KIBA and fine-tuned on GLASS achieved a test MSE of 0.57, while the same model trained on GLASS and fine-tuned on KIBA achieved a test MSE of 0.24. In both cases, fine-tuned models underperformed the original task-specific models.

These results indicate that while GNNs are capable of learning expressive representations for drug–target pairs, their ability to generalize is strongly influenced by the target diversity in the data set and the type of data split used. In particular, it is very difficult to train a well-performing model that would not be protein family specific. Based on its consistently superior performance across both data sets, the GIN_protein_–GIN_drug_ model was selected for all downstream analyses of explainability and biological relevance.

### Attribution Consistency across Methods

While basic gradient-based explainability methods such as Input × Gradient and guided backpropagation compute $\frac{\partial F\left(x\right)}{\partial x\left(i\right)}$ , these suffer from gradient saturation^63^ and violate axiomatic properties such as *sensitivity* (nonzero attribution for feature-impacting predictions) and *implementation invariance* (consistent attributions for functionally equivalent models).^48^ We therefore also implemented advanced techniques such as integrated gradients and gradient SHAP. To assess attribution consistency, we computed the intersection-overunion (IoU) for the top 50% most attributed atoms across all four explanation methods in the biological relevance test split, using the best-performing GINConv model.

For the GLASS data set, the average IoU score was 0.1636 ± 0.0904 for 30,922 drug-target pairs, while for the KIBA data set, the average IoU score was 0.15 ± 0.0930 for 33,236 drug-target pairs. This reflects a modest level of agreement among the four different attribution methods for both data sets (Figure 2). While standard benchmarks (like images or nonchemical text) often show favorable agreement among feature attribution approaches,^64^ applying them to complex graph data for molecular binding introduces massive variance. In our specific case, the main driver of the lower agreement is likely the DTI models themselves: because the models’ predictive accuracy is not yet perfect, the network itself lacks a highly constrained, single “golden path” for making its decisions. This wide, loose solution space allows different XAI strategies to capture disparate mathematical artifacts. Consequently, the level of agreement between the standalone XAI methods will intrinsically remain low unless the methods are ensemble-based or use biological sampling to filter out the noise. Only data points with nonzero overlap and available structural data in the test data set were selected for downstream analysis.

**Figure 2:**
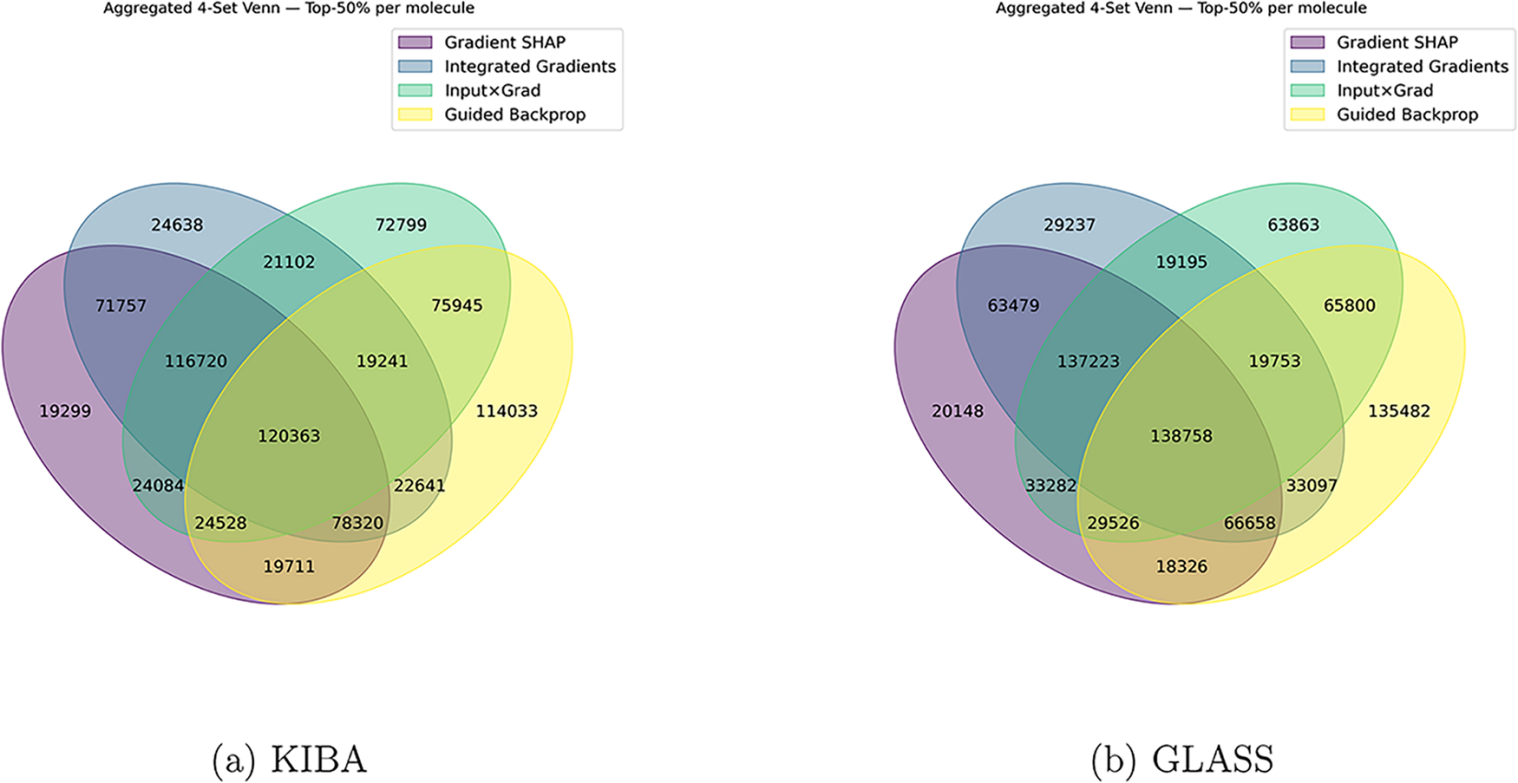
Four-set Venn diagrams for the KIBA (left) and GLASS (right) data sets. The diagrams display the exclusive counts of atoms identified by different subsets of the four explanation methods.

### Biological Relevance of Attributions

To determine whether the purely data-driven attributed atoms correspond to functionally important parts of the small molecules, we quantified their proximity to binding pocket residues in the three-dimensional (3D) structures of experimentally solved complexes (see the Supporting Information). In the GNN architectures employed in this study, the small molecules and protein targets are encoded entirely separately. As a result, the model possesses no prior knowledge of the 3D binding conformation or the contact interface between the ligand and the target; all atoms within the generic graph are treated equally, regardless of the interactions they might eventually form within the binding site. In the KIBA data set (759 experimentally solved complexes), attributed atoms are highly enriched in a very close proximity to the determined binding pocket annotated from KLIFs: at the 2 Å cutoff, nearly 76% of ligand atoms were marked as important by all four attribution methods. This enrichment declines as the distance grows, reaching 15% at 3 Å and 13% at 4 Å, as the number of ligand atoms within the cutoff increased faster than the number of attributed atoms (Figure 3a,c). In the GLASS data set (161 experimentally solved complexes), a similar trend was observed: at 2 Å, 33% of ligand atoms were attributed, i.e., marked as important, with enrichment decreasing to 15% at 3 Å and 11% at 4 Å (Figure 3b,d).

**Figure 3:**
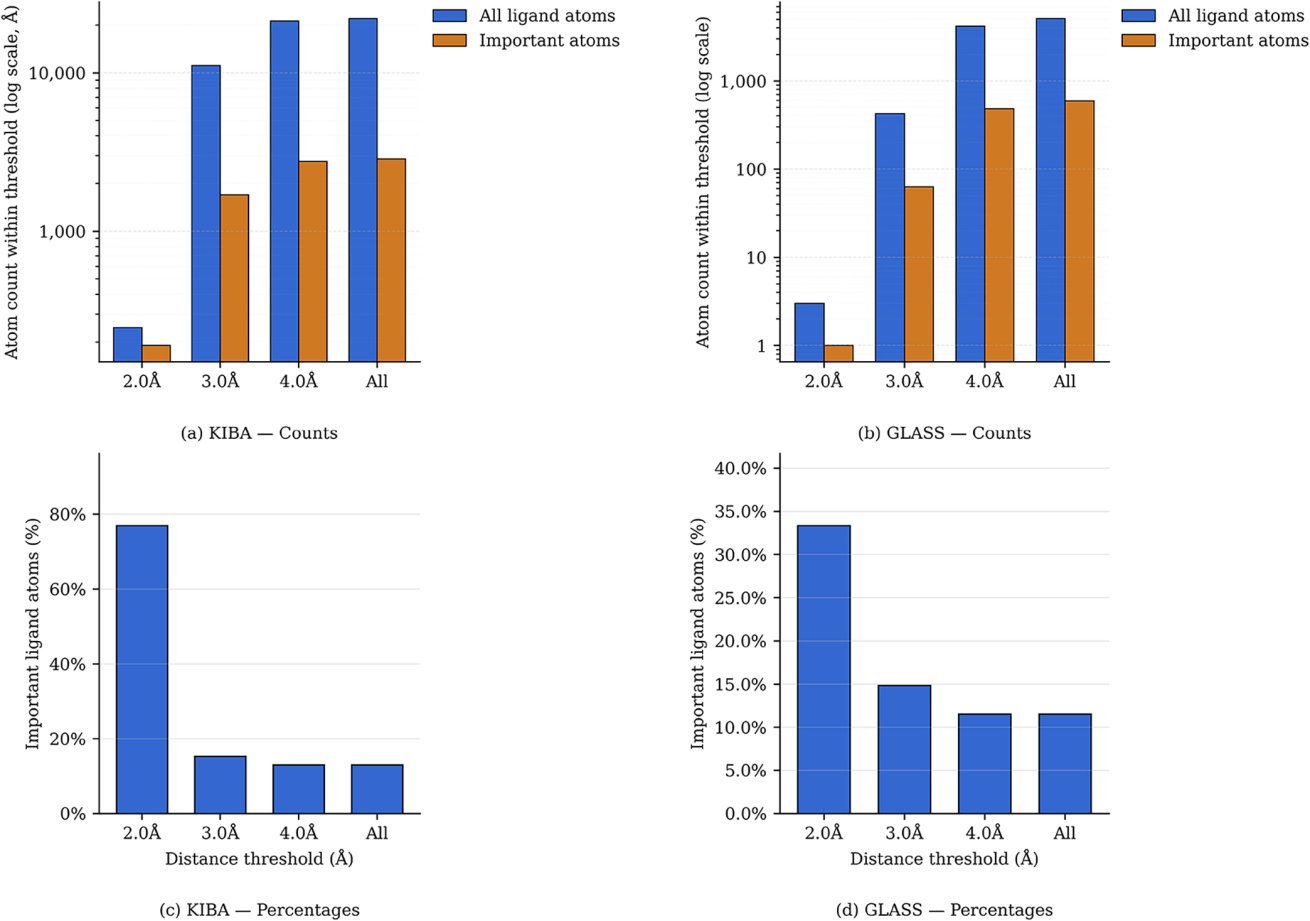
Comparison of atom counts and percentages across distance thresholds for the KIBA and GLASS data sets.

In summary, the consensus attributions, despite modest agreement between the methods, preferentially highlight atoms directly interacting with the protein pocket. This effect is more pronounced in the KIBA data set, where a larger number of experimentally solved complexes supports strong and reproducible enrichment at direct contacts. In GLASS, the enrichment is weaker but still evident, indicating that consensus attributions capture biologically meaningful ligand substructures, even in a smaller and more heterogeneous set of complexes.

For example, in the complex of mitogen-activated protein kinase 14 (MAPK14; UniProt accession Q16539) and the compound with the ChEMBL identifier CHEMBL328242, four of the eight attributed atoms are in direct contact with the protein (Figure 4a), and in the complex of endothelin receptor type B (EDNRB; UniProt accession P24530) and bosentan (ChEMBL identifier CHEMBL957) seven of the seven interact with the protein directly (Figure 4b).

**Figure 4:**
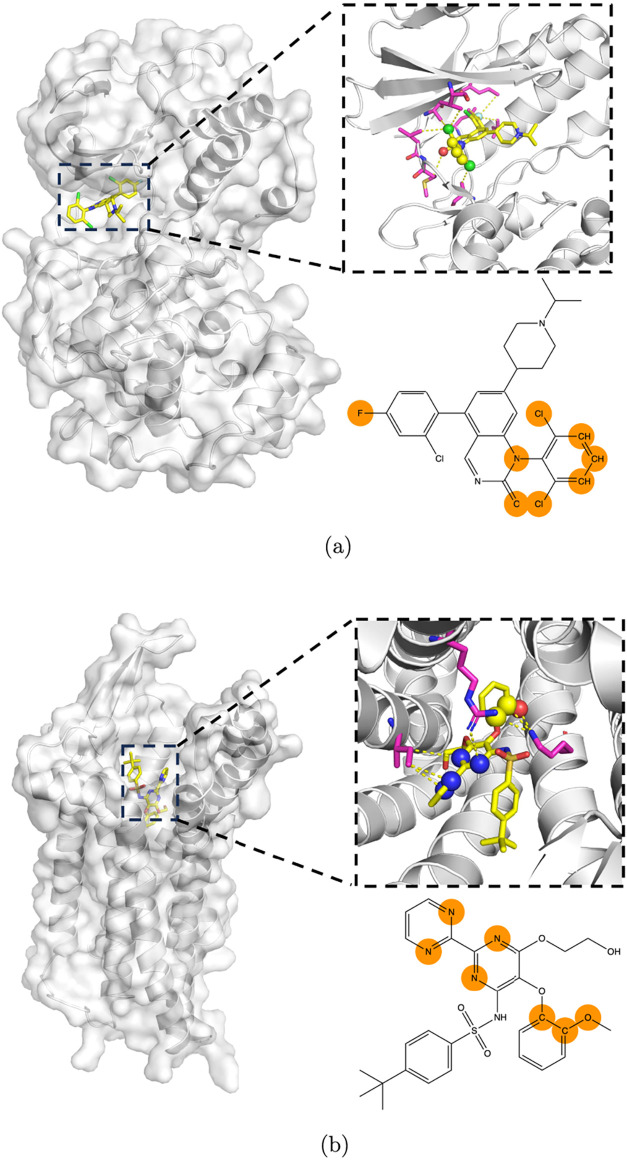
Example complexes from the GLASS and KIBA data sets with consensus-attributed atoms. Attributed atoms are shown as balls in the stick representation of the ligands. Contacting amino acid residues of the protein are shown in sticks as well and colored magenta. (a) KIBA: Mitogen-activated protein kinase 14 is associated with ligand CHEMBL328242. Structure from PDB entry 3GC7. (b) GLASS: Endothelin receptor type B with the ligand bosentan. Structure from the PDB entry 5XPR.

For 69 proteins from the KIBA data set and 43 proteins from the GLASS data set, several small molecules were observed to interact with the same protein. In these proteins, the attributed atoms of the different interactors were compared to assess consistency across ligands binding to the same protein. Three of the available ligands were chosen for the KIBA data set and GLASS data set, respectively. The corresponding protein–ligand complexes were overlaid, and the consensus-attributed atoms were examined to evaluate how well they mapped in three-dimensional space. For the KIBA data set, the overlay of three ligands with the ChEMBL identifiers CHEMBL2029678, CHEMBL2029688, and CHEMBL2031893 of the hepatocyte growth factor (HGF) receptor (UniProt accession P08581) revealed that the consensus-attributed atoms were consistently located near the DFG motif, a well-established regulatory element of the kinase active site (Figure 5a). Despite differences in their chemical scaffolds, the highlighted atoms converged within this conserved pocket region, suggesting that the explanation methods capture structural determinants that are central to kinase inhibition. The spatial alignment of attributed atoms across different ligands reinforces the view that the model recognizes ligand atoms in the proximity of activity-relevant pocket residues, implying the recognition of biologically meaningful motifs within the kinase binding site. As an example from the GLASS data set, we analyzed three ligands of the melatonin receptor type 1A (UniProt accession P48039): CHEMBL15060, agomelatine (CHEMBL10878), and iodomelatonin (CHEMBL289233). Most of the identified attributed atoms are positioned in close contact with the protein, indicating that the explanation methods emphasized ligand atoms likely to interact directly with the receptor, as seen in Figure 5b.

**Figure 5:**
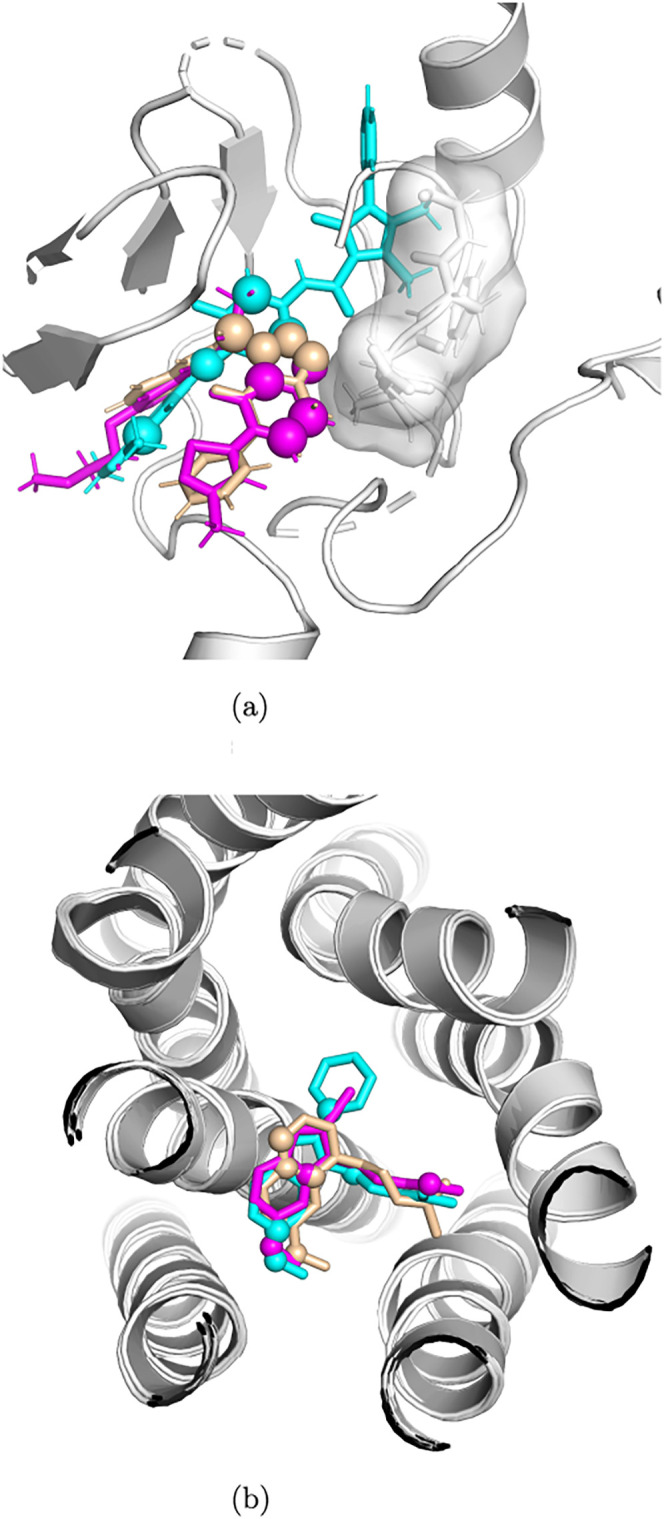
Example complexes from KIBA and GLASS with multiple ligands. The three-dimensional structures were overlaid based on aligning the protein molecules. Attributed atoms are shown as balls in the stick representation of the ligands and colored in wheat, magenta, and cyan, respectively. The DFG regions in kinases in plot (a) are shown as the surface representation. (a) Overlay of a KIBA sample with the same protein target but multiple binding ligands: The structures illustrated here have the following PDB identifiers: 4DEI, 4DEH, and 3U6H, which are colored magenta, wheat, and cyan, respectively. (b) Overlay of a GLASS sample with the same protein target but multiple binding ligands: The structures illustrated here have the following PDB identifiers: 6ME3, 6ME4, and 6ME5, which are colored cyan, magenta, and wheat, respectively.

To further assess the spatial consistency of attributions for different ligands bound to the same protein, we compared the pairwise distances between the centers of mass (COM) of consensus-attributed atoms against the pairwise distances between the COM of all ligand atoms (Figure 6) from individual ligands. This analysis revealed that the attributed atoms from different ligands are not necessarily colocated in proximity to one another. Using the pairwise distance between the COM of all ligand atoms as a benchmark, we found that the distances between the COMs of attributed atoms were consistently larger, indicating a lack of spatial convergence. We further analyzed the position of attributed atoms within individual ligands by measuring the distance from the COM of attributed atoms to the ligand’s total molecular COM, comparing it to the same measure for nonattributed atoms (Figure 7). The results indicate that consensus-attributed atoms are not centrally located but are instead enriched on the molecular periphery, consistent with their role at the protein–ligand interface. While the use of the COM has limitations—as it can be sensitive to the inclusion or exclusion of individual atoms in small sets, the overall trend is clear: attributed atoms across different ligands binding the same target are not necessarily colocated in a single, conserved spatial locus, but are consistently positioned at the ligand’s interaction surface.

**Figure 6:**
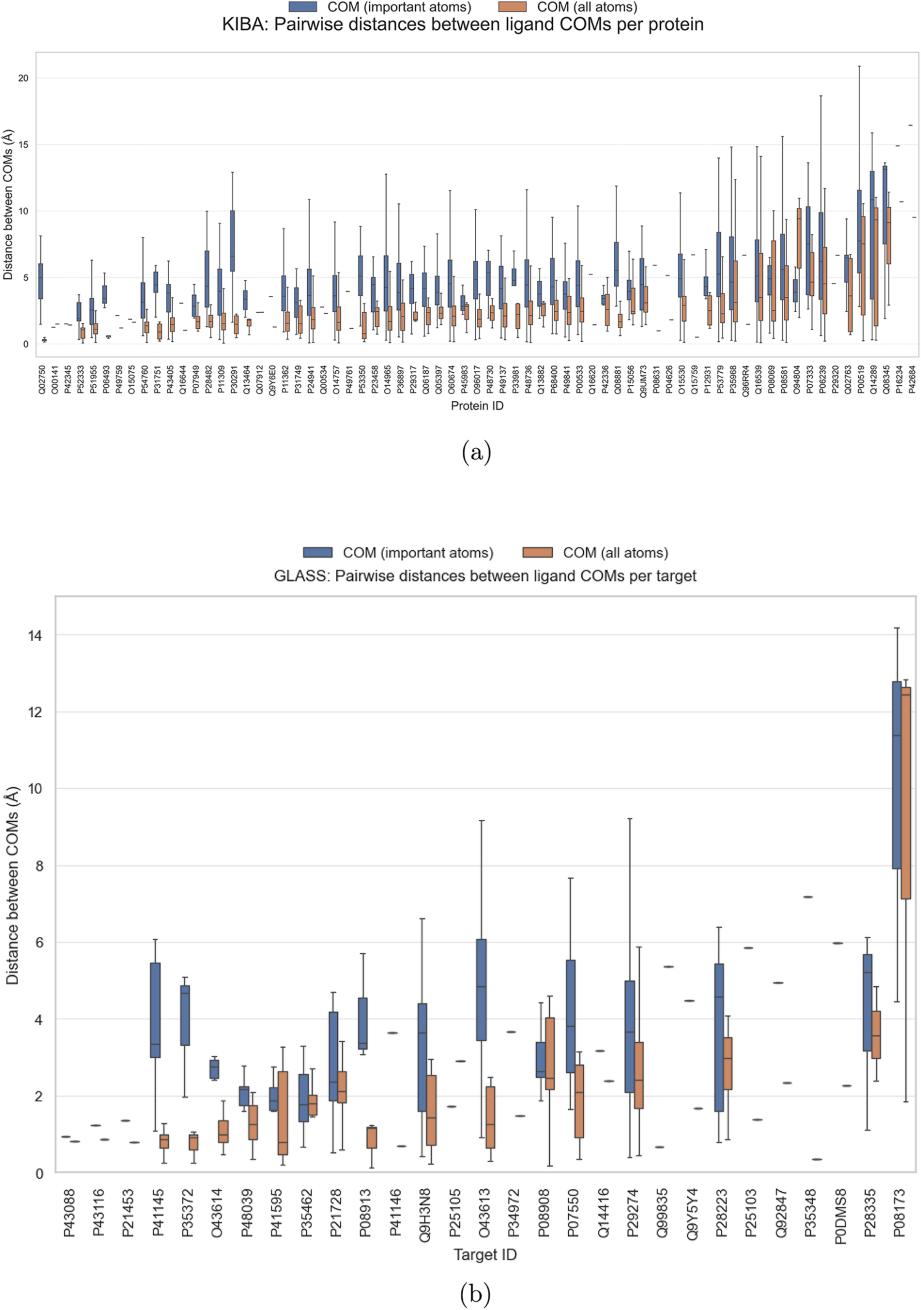
Comparison of pairwise center-of-mass (COM) distances for KIBA (a) and GLASS (b) data sets. The distances of attributed atoms are plotted in blue, and the distances of all ligands’ atoms are colored in orange. Means and standard deviations for different ligands binding the same protein are shown. (a) KIBA data set: Pairwise distance between COMs of attributed atoms from each ligand and that of COMs of whole ligand. (b) GLASS data set: Pairwise distance between COMs of attributed atoms from each ligand, and that of COMs of whole ligand.

**Figure 7:**
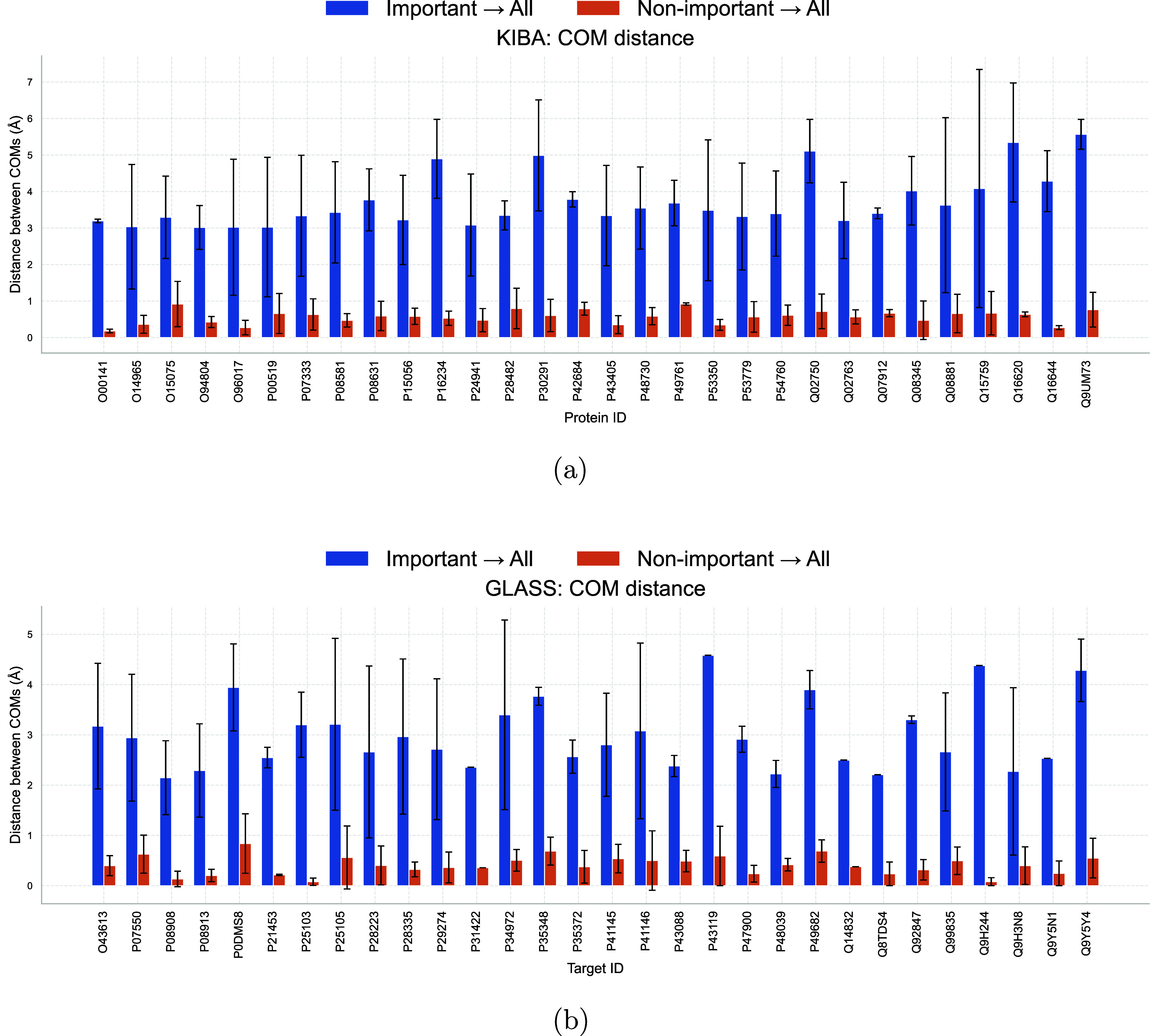
Comparison of center-of-mass (COM) distances between attributed atoms and whole ligands, vs nonattributed atoms and whole ligands, for KIBA (a) and GLASS (b) data sets. The distances of attributed atoms are plotted in blue, and nonattributed atoms are plotted in orange. Means and standard deviations for different ligands binding the same protein are shown. (a) KIBA data set: Distance between COM of attributed atoms and COM of whole ligand from individual ligand, and that of nonattributed atoms. (b) GLASS data set: Distance between COM of attributed atoms and COM of whole ligand from individual ligand, and that of nonattributed atoms.

## Limitations

Despite the promising insights offered by our consensus attribution framework, there are several limitations to our study. First, relying on an Intersection-over-Union (IoU) consensus approach to merge explanation methods inherently presents a 2-fold disadvantage. On one hand, preserving the most robust structural signals through intersection risks high sparsity; if attribution agreement is exceptionally low, the resulting consensus region may contain too few atoms to be practically useful, although the atoms reliably represent the interaction surface. On the other hand, this binary intersection approach inherently strips the continuous attribution weights, meaning that even when a highly populated consensus set is successfully identified, we lose the granular scoring necessary to rank those constituent atoms by their relative hierarchical importance. Second, there are inherent boundaries to the generalizability of our findings. This study rigorously tested and verified the biological plausibility of attributed atoms on two extensively studied protein families: protein kinases (KIBA data set) and G-protein-coupled receptors (GLASS data set). Although they display high target diversity internally and are highly relevant to clinical drug design, they represent only a subset of pharmacological target classes, meaning that our results cannot definitively be generalized to entirely different classes (e.g., highly flexible intrinsically disordered proteins). Even for these data sets, our models demonstrate only modest performance, especially for challenging cold splits, emphasizing their limited generalizability. Future work will be required to expand this structural validation globally across massive, multiclass databases (e.g., BindingDB^65^). Finally, relying on the random split to assign atom attributions is not optimal since this split introduces the highest data leakage in the training process. In this study, this choice was dictated by poor performance on low-leakage splits. Nevertheless, this analysis demonstrates that simple attribution techniques are capable of providing biologically meaningful insights, which we believe will also be the case with more performant models in low-leakage training scenarios.

## Conclusion

In this study, we investigated the explainability of graph-neuron-network-based drug-target interaction models by applying four attribution techniques: Input × Gradient, Guided Backpropagation, Integrated Gradients, and Gradient SHAP on two benchmark data sets, protein kinases and their inhibitors (KIBA) and G-protein-coupled receptors and their ligands (GLASS). Our analysis revealed that the different methods often converged on overlapping subsets of ligand atoms, suggesting a degree of robustness across explanation techniques. These atoms also tend to directly contact the corresponding proteins, suggesting that they indeed play an important role in protein–ligand interactions. Moreover, when ligands binding to the same protein were overlaid, the attributed atoms, although located in the protein-contacting parts of the ligands, often contact different sites in the binding pocket, indicating the importance of different pharmacophores.

Overall, our results suggest that post hoc attribution methods can provide chemically meaningful insights into model predictions, offering a step toward interpretable and reliable applications of deep learning in drug discovery. Future work could expand on these findings by exploring additional explanation families and extending the analysis to larger and more diverse data sets.

## Supplementary Material











## Data Availability

We provide our evaluation code and consensus analysis pipelines publicly to enable reproducibility and accelerate the development of trustworthy models at https://github.com/kalininalab/LigandXai. The package RINDTI is available at https://github.com/kalininalab/rindti. The cleaned KIBA and GLASS data sets are available in the Supporting Information.

## Notes

Calculation of the KIBA score (Note 1); detailed model configuration, implementation details of the graph neural network models used in our experiments (Note 2) (PDF)
Processed KIBA data set used for model training (File S3) (CSV)
Processed GLASS data set used for model training (File S4) (CSV)
List of all protein−ligand complexes from the KIBA data set, including protein target identifiers, ligand identifiers, and complex structure IDs, that were used for the structural proximity analysis (File S1) (CSV)
List of all protein−ligand complexes from the GLASS data set, including protein target identifiers, ligand identifiers, and complex structure IDs, that were used for the structural proximity analysis (File S2) (CSV)
