# Supplementary material for: Explainability Methods from Machine Learning Detect Important Drugs’ Atoms in Drug-Target Interactions
Source: J Chem Inf Model. 2026 Apr 15;66(8):4947–61. doi: 10.1021/acs.jcim.6c00037 (PMC13126640; doi:10.1021/acs.jcim.6c00037)
Supplement: Supplementary file 1 [file ci6c00037_si_001.pdf]

Supporting Information

Explainability methods from machine learning detect important drugs' atoms in drug-target interactions

Mrinal Mahindran,[*,†,‖] Qingyuan (Chingyuen) Liu,[*,†,‡,¶,‖] Vishak Madhwaraj Kadambalithaya,[†,‡] and Olga V Kalinina[*,†,‡,§]

†*Center for Bioinformatics, Saarland University, Saarbrücken*

‡*Helmholtz Institute for Pharmaceutical Research Saarland (HIPS), Helmholtz Centre for Infection Research (HZI), Saarbrücken*

¶*International Max Planck Research School on Trustworthy Computing, Saarbrücken*

§*Medical Faculty, Saarland University, Homburg*

‖ - *equal contribution*

E-mail: mrmr00001@stud.uni-saarland.de; qingyuan.liu@helmholtz-hips.de; olga.kalinina@helmholtz-hips.de

Supplementary File S1: KIBA structural dataset

File:`final_kiba.csv`

This file lists all protein–ligand complexes from the KIBA dataset that were used for the proximity analysis. Each entry includes the protein target identifier, drug (ligand) identifier, and the corresponding structure ID of the co-crystallised complex. These data define the

subset of experimentally resolved structures used to evaluate the spatial correspondence between model-attributed atoms and binding pocket residues.

Supplementary File S2: GLASS structural dataset

File:`final_glass.csv`

This file provides analogous information for the GLASS dataset, including protein target identifiers, ligand identifiers, and complex structure IDs used for the proximity analysis. Together, these files document the structural basis of the datasets employed in the biological relevance evaluation.

Supplementary File S3: KIBA training dataset

File:`KIBA.csv`

This file contains the processed KIBA dataset used for model training. Each entry corresponds to a drug–target interaction pair and includes the protein target identifier, ligand identifier, and the associated binding affinity value (KIBA score). The dataset represents the full set of samples used to train and evaluate the graph-based models in this study.

Supplementary File S4: GLASS training dataset

File:`GLASS.csv`

This file contains the processed GLASS dataset used for model training. Similar to the KIBA dataset, each entry includes protein target identifiers, ligand identifiers, and the corresponding binding affinity values (pKi). This dataset was used to train and evaluate the models on GPCR-related interactions.

Supplementary Note 1: KIBA Score

Calculation of the KIBA score.

Supplementary Note 2: Detailed Model Configuration

Implementation details of the graph neural network models used in our experiments.

Supporting Information - Supplementary notes
Explainability methods from machine learning detect important drugs' atoms in drug-target interactions

Mrinal Mahindran,[*,†,‖] Qingyuan (Chingyuen) Liu,[*,†,‡,¶,‖] Vishak Madhwaraj Kadambalithaya,[†,‡] and Olga V Kalinina[*,†,‡,§]

†*Center for Bioinformatics, Saarland University, Saarbrücken*

‡*Helmholtz Institute for Pharmaceutical Research Saarland (HIPS), Helmholtz Centre for Infection Research (HZI), Saarbrücken*

¶*International Max Planck Research School on Trustworthy Computing, Saarbrücken*

§*Medical Faculty, Saarland University, Homburg*

‖ - *equal contribution*

E-mail: mrmr00001@stud.uni-saarland.de; qingyuan.liu@helmholtz-hips.de; olga.kalinina@helmholtz-hips.de

Supplementary Note 1: KIBA Score

The KIBA score integrates heterogeneous kinase inhibitor bioactivity measurements (IC_{50}, K_i, and K_d) into a single unified metric. To harmonise the different assay formats, a model-based adjustment was introduced to rescale K_i and K_d using IC_{50}, thereby increasing the consistency between bioactivity types.[1]

The adjustment formulas are:

$$K_i^{\text{adj}} = \frac{\text{IC}_{50}}{1 + L_i\left(\frac{\text{IC}_{50}}{K_i}\right)},$$

$$K_d^{\text{adj}} = \frac{\text{IC}_{50}}{1 + L_d\left(\frac{\text{IC}_{50}}{K_d}\right)},$$

where L_i and L_d are the parameters that determine the weights of IC_{50} in the model-based adjustments for K_i and K_d. The final KIBA score for each drug–target pair is defined as:

$$\text{KIBA} = \begin{cases} K_i^{\text{adj}}, & \text{if IC}_{50} \text{ and } K_i \text{ are available} \\ K_d^{\text{adj}}, & \text{if IC}_{50} \text{ and } K_d \text{ are available} \\ \frac{K_i^{\text{adj}} + K_d^{\text{adj}}}{2}, & \text{if all three values are available.} \end{cases}$$

This integrated score provides a harmonised quantitative representation of the inhibitor bioactivity and improves the consistency between different assay types.

Supplementary Note 2: Detailed Model Configuration

This section provides additional implementation details of the graph neural network models used in our experiments.

Graph Representation

Drug molecules are represented as molecular graphs where nodes correspond to atoms and edges correspond to chemical bonds. Each atom is encoded using a 14-dimensional one-hot representation of atom type. Proteins are represented as residue-level graphs where nodes correspond to amino acid residues. Each residue is encoded using a 20-dimensional one-hot representation corresponding to the standard amino acid alphabet.

Encoder Architecture

Our framework uses a dual-encoder architecture consisting of one graph encoder for the drug and one graph encoder for the protein. Each encoder contains four message-passing layers: one input layer, two intermediate layers, one output layer. After message passing, node embeddings are aggregated using global mean pooling to obtain graph-level representations.

We evaluated six graph neural network architectures as encoders: GINConv,[2] GATConv,[3] GraphSAGE,[4] ChebConv,[5] FiLMConv,[6] and Transformer-GNN.[7]

Unless otherwise stated, the same encoder type is used for both the drug and protein branches.

Hidden Dimensions and Attention Heads

The hidden dimensionality depends on the encoder architecture: GINConv, GraphSAGE, ChebConv, and FiLMConv use a hidden dimension of 256, and GATConv and Transformer-GNN use a hidden dimension of 128. For attention-based architectures, the number of heads is configured as follows: GATConv uses 4 attention heads in the input and intermediate layers and 1 head in the output layer; Transformer-GNN uses 2 attention heads in the input and intermediate layers and 1 head in the output layer.

Prediction Network

The graph-level drug and protein embeddings are concatenated and passed to a multilayer perceptron (MLP) for affinity prediction. The prediction network consists of a four-layer MLP with a hidden dimension 256: $d_{in} \to 256 \to 256 \to 256 \to 1$, where d_{in} is the concatenated embedding dimension (either 256 or 512 depending on the encoder). Each hidden layer is followed by ReLU activation and dropout with probability 0.2. The final layer outputs a single scalar corresponding to the predicted binding affinity.

Training Details

Models are trained using the mean squared error (MSE) loss and the Adam optimizer. Due to memory requirements of attention-based models, different batch sizes are used: GINConv, GraphSAGE, ChebConv, and FiLMConv use batch size 256; and GATConv and Transformer-GNN use batch size 128.

For the encoder comparison experiments (Table 5), each model was trained using five different random seeds on the random split, and the reported results correspond to the mean ± SEM across seeds. For the split comparison experiments (Table 3) and the encoder-combination experiments (Table 4), models were trained using a single run per configuration.

Table S1: Summary of model architecture and training configuration used in the experiments.

Component	Specification
Drug node features	14 (atom type one-hot encoding)
Protein node features	20 (amino acid one-hot encoding)
Number of GNN layers	4 (input + 2 intermediate + output)
Pooling method	Global mean pooling
Hidden dim (GIN/SAGE/Cheb/FiLM)	256
Hidden dim (GATConv/Transformer)	128
GATConv attention heads	4 (input/intermediate), 1 (output)
Transformer attention heads	2 (input/intermediate), 1 (output)
Prediction head	4-layer MLP ($d_{in} \rightarrow 256 \rightarrow 256 \rightarrow 256 \rightarrow 1$)
Activation	ReLU
Dropout	0.2
Loss function	Mean Squared Error (MSE)
Optimizer	Adam
Batch size (GIN/SAGE/Cheb/FiLM)	256
Batch size (GATConv/Transformer)	128
Max training epochs	500
Early stopping patience	50
Random seeds	5 (encoder comparison experiments)

References

(1) Tang, J.; Szwajda, A.; Shakyawar, S.; Xu, T.; Hintsanen, P.; Wennerberg, K.; Aittokallio, T. Making sense of large-scale kinase inhibitor bioactivity data sets: a comparative and integrative analysis. *Journal of Chemical Information and Modeling* **2014**, *54*, 735–743.

(2) Brossard, R.; Frigo, O.; Dehaene, D. Graph convolutions that can finally model local structure. 2021; `https://arxiv.org/abs/2011.15069`.

(3) Veličković, P.; Cucurull, G.; Casanova, A.; Romero, A.; Liò, P.; Bengio, Y. Graph Attention Networks. 2018; `https://arxiv.org/abs/1710.10903`.

(4) Hamilton, W. L.; Ying, R.; Leskovec, J. Inductive Representation Learning on Large Graphs. 2018; `https://arxiv.org/abs/1706.02216`.

(5) He, M.; Wei, Z.; Wen, J.-R. Convolutional Neural Networks on Graphs with Chebyshev Approximation, Revisited. 2024; `https://arxiv.org/abs/2202.03580`.

(6) Brockschmidt, M. GNN-FiLM: Graph Neural Networks with Feature-wise Linear Modulation. 2020; `https://arxiv.org/abs/1906.12192`.

(7) Yun, S.; Jeong, M.; Kim, R.; Kang, J.; Kim, H. J. Graph Transformer Networks. 2020; `https://arxiv.org/abs/1911.06455`.
